# Correction: Multi-view classification with convolutional neural networks

**DOI:** 10.1371/journal.pone.0250190

**Published:** 2021-04-08

**Authors:** The PLOS ONE Staff

## Notice of republication

This article was republished on March 26, 2021 to correct an error in the Author Contributions. The publisher apologizes for the error. Please download this article again to view the correct version. The originally published, uncorrected article and the republished, corrected article are provided here for reference.

## Supporting information

S1 File Originally published, uncorrected article. (PDF)

S2 File Republished, corrected article. (PDF)
