# Supplementary material for: Correction: Multi-view classification with convolutional neural networks
Source: PLoS One. 2021 Apr 8;16(4):e0250190. doi: 10.1371/journal.pone.0250190 (PMC8031445; doi:10.1371/journal.pone.0250190)
Supplement: S1 File — (PDF) [file pone.0250190.s001.pdf]

PLOS ONE

RESEARCH ARTICLE

# Multi-view classification with convolutional neural networks

**Marco Seeland*, Patrick Mäder**

Institute for Computer and Systems Engineering, Technische Universität Ilmenau, Ilmenau, Germany

* marco.seeland@tu-ilmenau.de

Check for updates

OPEN ACCESS

**Citation:** Seeland M, Mäder P (2021) Multi-view classification with convolutional neural networks. PLoS ONE 16(1): e0245230. https://doi.org/10.1371/journal.pone.0245230

**Editor:** Robertas Damaševičius, Politechnika Slaska, POLAND

**Received:** June 17, 2020

**Accepted:** December 26, 2020

**Published:** January 12, 2021

**Copyright:** © 2021 Seeland, Mäder. This is an open access article distributed under the terms of the Creative Commons Attribution License, which permits unrestricted use, distribution, and reproduction in any medium, provided the original author and source are credited.

**Data Availability Statement:** - CompCars dataset is available from http://mmlab.ie.cuhk.edu.hk/datasets/comp_cars/index.html - PlantCLEF dataset is available from https://www.imageclef.org/lifeclef/2016/plant - AntWeb data is available from the online database www.antweb.org.

**Funding:** We are funded by the German Federal Ministry for the Environment, Nature Conservation, Building and Nuclear Safety (BMUB) grants: 3514685C19, 3519685; the German Ministry of Education and Research (BMBF) grants: 01LC1319, 01IS20062; the Thuringian Ministry for Environment, Energy and Nature Conservation

## Abstract

Humans' decision making process often relies on utilizing visual information from different views or perspectives. However, in machine-learning-based image classification we typically infer an object's class from just a single image showing an object. Especially for challenging classification problems, the visual information conveyed by a single image may be insufficient for an accurate decision. We propose a classification scheme that relies on fusing visual information captured through images depicting the same object from multiple perspectives. Convolutional neural networks are used to extract and encode visual features from the multiple views and we propose strategies for fusing these information. More specifically, we investigate the following three strategies: (1) fusing convolutional feature maps at differing network depths; (2) fusion of bottleneck latent representations prior to classification; and (3) score fusion. We systematically evaluate these strategies on three datasets from different domains. Our findings emphasize the benefit of integrating information fusion into the network rather than performing it by post-processing of classification scores. Furthermore, we demonstrate through a case study that already trained networks can be easily extended by the best fusion strategy, outperforming other approaches by large margin.

## Introduction

Convolutional neural networks (CNNs) represent the state of the art in computer vision and perform on par or even better than humans in manifold tasks [1, 2]. CNNs have especially been demonstrated to yield great potential for fine-grained classification problems [3–6]. However, there are fine-grained classification problems where a single image does not yield sufficiently discriminative information for accurate classification and a continuous demand for better classifier performance exists. In this paper, we study multi-view classification in the area of machine learning as one way to improve classification performance. Thereby, **view** is meant literal, i.e., each view is a distinct image displaying the same **object** instance or part of it from a certain perspective. Images of the same object from different views form an image **collection** (cp. Fig 1).

Classification based on multi-view collections is especially relevant for objects characterized by high inter-class similarity and intra-class variability, hence, for fine-grained classification problems where different views of the same object are expected to provide complementary

grant: 68678; and the Stiftung Naturschutz Thüringen (SNT) grant: SNT-082-248-03/2014. We acknowledge support for the publication costs by the Open Access Publication Fund of the Technische Universität Ilmenau.

**Competing interests:** The authors have declared that no competing interests exist.

information. Multi-view classification is inspired by humans' behavior, e.g., a botanist observing various traits of an unknown plant to be identified. Plant species identification also represents a typical use-case for multi-view classification (cp. Fig 1), where certain species can hardly be distinguished by their flowers alone, but require additional information, e.g., regarding their leaves [3].

In general, multi-view CNNs seek to combine useful information from different views so that more comprehensive representations may be learned yielding a more effective classifier [7, 8]. However, it remains unclear which model topology and fusion operation are most effective for information fusion. We propose and evaluate three strategies for fusing visual information from different views of an object instance at different stages of the prediction process. For the strategy that fuses information within a CNN's feature extraction, we further investigate classifier performance with respect to depth of fusion. To the best of our knowledge, this study represents the first systematic investigation of this problem. Acquiring and labeling collection data requires vast efforts [9]. Within the past years, several collection-based datasets were acquired by researchers or citizen-science and community-driven initiatives enabling us to investigate multi-view classification on large real-world datasets from three different domains: (1) plants [10], (2) car brands and models [11], and (3) insects (the Formicidae ants genera) [5]. With emerging consumer-level technologies, e.g., 3D scanning and visual odometry from

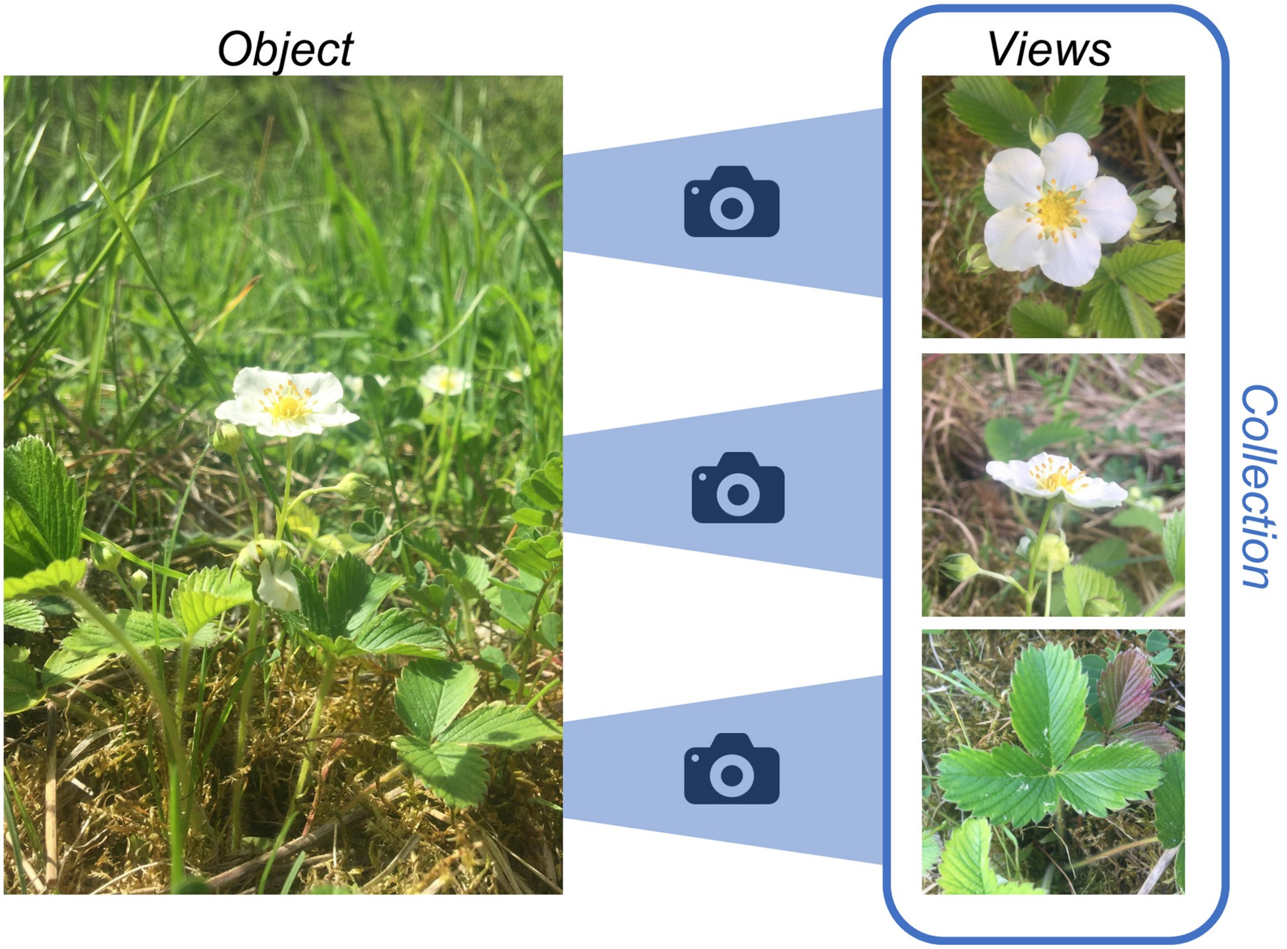


**Fig 1. A collection of images is composed of multiple views depicting the same object instance from different perspectives.**

https://doi.org/10.1371/journal.pone.0245230.g001

image series [12–15], we expect an increasing number of collection-based datasets and rising interest in multi-view classification.

The contributions of our study are as follows:

- *Overview*: we provide a concise overview on relevant previous studies proposing multi-view classification.
- *Fusion strategies*: we derive three potential fusion strategies for CNN-based multi-view classification.
- *Systematic evaluation*: we conduct a systematic evaluation on three datasets from different domains.
- *Usage scenario*: we demonstrate the ease of applying the best performing fusion strategy on an exemplary use case resulting in considerably increased classification accuracy.

## Related work

Supervised training of deep neural networks requires a vast amount of labeled data. Available image datasets typically depict objects solely by single images rather than a collection of views. This limited availability of collection data and the need for representative datasets for training efficient deep models results in little research been conducted towards multi-view object classification with deep learning. Table 1 provides an overview of previous work that we deem relevant with respect to multi-view object classification. Per study, we list the application domain, the utilized features, and the applied fusion methods and discuss selected methods in detail below.

Closest to multi-view object classification is 3D object recognition. There, Su et al. introduce a multi-view CNN (MVCNN) architecture that aggregates information from multiple 2D views of computer-designed 3D objects into single and compact representations [11]. For each view, i.e., each image rendered from a 3D model of an object, they use the output of the penultimate fully-connected layer of a pre-trained VGG-M model as feature map. The authors fuse twelve views by an element-wise maximum operation across all feature maps and found slightly increased accuracy when using 80 views. Feng et al. extend the MVCNN by introducing a view-grouping module [16]. Rather than equally fusing information from different views by a maximum operation, they group views based on discrimination scores determined by

**Table 1. Overview of previous work utilizing multi-view classification.**

| Study | Application domain | Fused features* | Fusion method | Performance | |
|---|---|---|---|---|---|
| | | | | metric | score |
| Su et al. [11] | 3D shape classification (ModelNet40) | fully-connected (8 × 4096) | maximum | acc. | 90.1% |
| Feng et al. [16] | 3D shape classification (ModelNet40) | conv feature maps (8 × 2048) | weighted sum | acc. | 93.1% |
| Lin et al. [17] | 3D fingerprint matching | fully-connected (3 × 128) | concatenation | acc. | 99.89% |
| Wang et al. [18] | RGB-D Object classification (RGB-D Objects) | fully-connected (2 × 128) | learned transformation | acc. | 88.5% |
| Do et al. [19] | Image classification (PlantCLEF2015 selection) | class scores (2 × 50) | product | acc. | 89.8% |
| Lee et al. [20] | Image classification (PlantCLEF2015 selection) | fully-connected ($N$ × 4096) | gated recurrent unit | acc. | 74.5% |
| Setio et al. [21] | Pulmonary nodule detection (LIDC-IDRI selection) | fully-connected (9 × 16) | concatenation | AUC | 99.3% |
| Dolata et al. [22] | Grain classification | class scores (2 × 7) | sum | acc. | 97.7% |
| Barbosa et al. [23] | Crop yield regression | fully-connected (16 × 1) | concatenation | MSE | 0.7 |
| Geras et al. [24] | Breast cancer screening | fully-connected (4 × 256) | concatenation | AUC | 73.3% |

* Numbers in brackets are (*no. of views* × *no. of dimensions*)

https://doi.org/10.1371/journal.pone.0245230.t001

auxiliary fully-connected layers. The fused feature map is then computed as weighted average of the grouped views showing slightly improved classification accuracy compared to MVCNN. Also, Geras et al. proposed a fusion architecture inspired by the MVCNN. They concatenated view-specific feature vectors to a combined feature vector that is fed into a fully connected layer for breast cancer screening [24]. Feature vector concatenation was also used by Lin et al. [17], who evolve these 3D shape classification approaches for 3D fingerprint recognition.

Wang et al. [18] fuse color and depth information for RGB-D object recognition. They pre-train two CNNs on color and depth images separately and then fuse the resulting feature vectors, i.e., the outputs of the penultimate fully-connected layers, by transformation to a joined representation containing modal-specific parts and a shared common part via regression. The method introduces additional parameters requiring manual optimization, e.g., the percentage of shared features.

Feichtenhofer et al. investigate various ways of fusing CNN feature maps both spatially and temporally [25]. They performed fusion by stacking the output of two network branches and subsequent $1 \times 1$ convolution for modeling the correspondence between the branches. They found that fusing feature maps of convolutional layers is more informative than fusion at the softmax layer.

Do et al. [19] perform plant species identification using multi-organ collections. The authors fine-tune an AlexNet and fuse the softmax scores of image pairs by sum-, product-, or max-rule. They found the product-rule to yield the highest classification accuracy. Furthermore, a product-rule fusion with scores of Support Vector Machines trained on the concatenated score vectors slightly increased accuracy. Lee et al. [20] propose a combination of convolutional and recurrent neural network for multi-organ based plant identification. They treat different views as image sequences ordered in terms of depicted plant organs. They model dependencies between different views by concatenating the bi-directional output of a Gated Recurrent Unit.

Setio et al. [21] evaluate three multi-view CNN-based fusion approaches for Pulmonary nodule detection in Computer Tomography images. Nine candidate patches from specific views are analyzed by corresponding network branches. The investigated fusion approaches are: (a) product-rule score fusion of separately trained CNNs, (b) concatenating the output of the penultimate fully-connected layers, followed by classification, and (c) fusion of manually grouped patches followed by product-rule based fusion of group scores. The authors found the concatenation approach to yield the best detection performance. Dolata et al. [22] also evaluate different fusion approaches for visual inspection of grains from two perspectives. They found sum-based score fusion to yield the highest classification accuracy.

Also Barbosa et al. investigated different strategies for fusing agronomy related maps for crop yield prediction [23]. They compared feeding stacked inputs through a multi-channel 2D or 3D CNN, concatenation of flattened convolution feature maps, and concatenation of the output of one single neuron for every branch. On a dataset gathered from nine corn fields, they found the feature concatenation strategy to achieve 26% less error compared to the stacked multi-channel 2D CNN.

In conclusion, previous studies demonstrate the feasibility and potential of multi-view classification for individual problems. However, we argue that a systematic study is required to substantiate findings in the following directions:

- *Fusing methods*: mostly element-wise sum or maximum operations have been studied for fusing CNN feature maps from multiple views for the purpose of classification. Correspondence between multiple views is thereby lost, while fusion by concatenation or convolution were found to efficiently model correspondences between different views for other learning

tasks. Comparative evaluations of different strategies for image classification are either missing or yield contradicting results.

- *Domains*: multi-view classification is mainly performed on domain-specific data, e.g., rendered images of 3D models and computer tomography scans. Results may not generalize well to other domains.
- *Scale*: utilized datasets for multi-view classification studies are small compared to current single view classification studies.

Combining all vacancies from above, we argue that a systematic evaluation of different fusion strategies on datasets from different domains is required.

## Fusion strategies for multi-view image classification

The task of image classification is inferring to which of $k$ categories an input image $\boldsymbol{x} \in \boldsymbol{X}$ belongs to. Thereby, a classifier is an approximated function $f$: $\boldsymbol{x} \rightarrow \{1, \ldots, k\}$ that maps the input $\boldsymbol{x}$ to its corresponding class label $y \in \{1, \ldots, k\}$. The default choice for image classifier are CNN [1, 2]. In this work, we exemplarily use widely applied ResNet-50 CNN [26] backbone network and extend it to multi-view classification by the strategies explained below. We chose the ResNet architecture due to its repetitive building blocks allowing to easily investigate the effect of varying depth of the view-fusion layer within the network. However, we argue that our results are transferable to other architectures as the alterations introduced by view-fusion do not depend on the underlying CNN architecture. We exemplify this statement by extending a NASNet-A network by multi-view fusion within Section *Application Scenario: Plant Species Identification*.

Next, we describe the different strategies for extending such single-view CNN architectures into multi-view architectures. We use the fact that images $\boldsymbol{x}$ are organized in terms of collections $\boldsymbol{X}_V$, where each collection contains a number $n_V$ of distinct views. Hence, we reformulate the classification task as

$$f : \boldsymbol{X}_V \rightarrow \{1, \ldots, k\} \mid \boldsymbol{X}_V = \{\boldsymbol{x}^{(1)}, \boldsymbol{x}^{(2)}, \ldots, \boldsymbol{x}^{(n_V)}\}, \quad (1)$$

where $\boldsymbol{x}^{(v)}$ represents one image from a specific view $v \in \{1, \ldots, n_V\}$. Each image $\boldsymbol{x}^{(v)}$ is processed by a separate branch of the same CNN. Following the literature discussed in Section *Related Work*, we systematically order the fusion strategies into **early**, **late**, and **score fusion**.

In **early fusion**, convolutional feature maps from the different CNN branches are stacked and subsequently processed together. In contrast, **late fusion** relies on aggregating the output of the last layer before the classification layer, or, in case of multiple fully connected layers at the top, the classification block, as latent representation. At last, **score fusion** is based on element-wise aggregation of the softmax classification scores per branch. Fig 2 provides an overview of the investigated view-fusion strategies and the related aggregation operations.

For early and late fusion, the original CNN is split into two parts, $NN_1$ and $NN_2$, and extended by a view fusion layer between both parts. Each view $v$ is forward propagated through a separate branch, which is a duplicate of $NN_1$. The layer weights of all branches are shared in order to limit the number of network parameters. After forward propagating an input image $\boldsymbol{x}^{(v)}$, the output of each branch is an intermediate representation $\boldsymbol{z}^{(v)} = NN_1(\boldsymbol{x}^{(v)})$. The task of the view fusion layer vfl is to form an aggregated representation $\hat{\boldsymbol{Z}} = \mathrm{vfl}(\boldsymbol{z}^{(1)}, \ldots, \boldsymbol{z}^{(n_V)})$ which is further processed by $NN_2$.

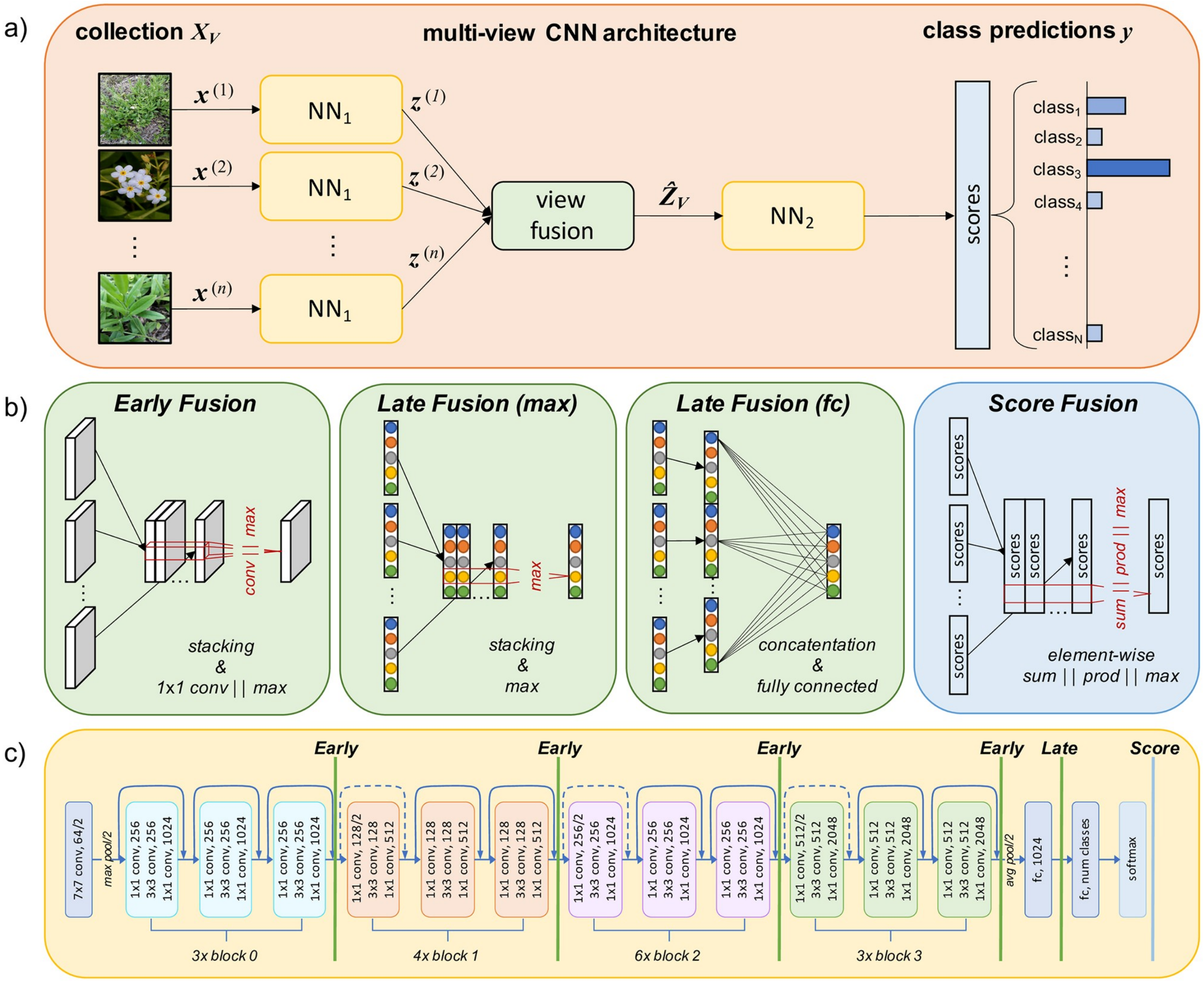


**Fig 2. Considered multi-view fusion strategies: (a) general architecture of a deep multi-view CNN; (b) investigated fusion strategies; and (c) fusion strategies mapped onto the ResNet-50 architecture.** Vertical lines mark the insertion of a view-fusion layer.

https://doi.org/10.1371/journal.pone.0245230.g002

## Early fusion

The forward propagation of an image $\boldsymbol{x}^{(v)}$ through each branch of $\mathrm{NN}_1$ yields a convolutional feature map

$$\boldsymbol{z}^{(v)} = \mathrm{NN}_1(\mathbf{x}^{(v)}) \in \mathbb{R}^{H\times W\times D}, \tag{2}$$

where $H$, $W$, and $D$ are the height, width, and depth (number of channels) of $\mathbf{h}^{(v)}$. A view fusion layer aggregates all feature maps of all $n_V$ branches into a stacked feature map

$$\boldsymbol{Z}_V = [\boldsymbol{z}^{(1)}, \boldsymbol{z}^{(2)}, \ldots, \boldsymbol{z}^{(n_V)}] \in \mathbb{R}^{H\times W\times Dn_V}. \tag{3}$$

$\mathrm{NN}_2$ consists of the remaining layers of the original CNN. Since the original CNN was pre-trained on single images, $\mathrm{NN}_2$ expects inputs of dimension $H \times W \times D$. Hence, the depth $Dn_V$ of the stacked feature maps $\boldsymbol{Z}_V$ has to be reduced to the depth $D$ of a single-view feature map. We consider two different approaches for depth reduction: (1) **early fusion (max)**: max-

pooling of the stacked feature map across the $n_V$ views; and (2) **early fusion (conv)**: $1 \times 1$ convolution across the depth of the stacked feature maps.

The max-pooling operation in **early fusion (max)** computes at each spatial position and channel $H \times W \times D$ in the stacked feature map $\mathbf{Z}_V$ the maximum value

$$\hat{\mathbf{Z}}_V = \max_v \mathbf{Z}_V \tag{4}$$

across the feature maps of all $n_V$ views. The result is a fused feature map of dimension $H \times W \times D$ in which correspondence between the different views is lost.

In **early fusion (conv)**, we replace the max-pooling operation by a $1 \times 1$ convolution operation as inspired by "Network-in-Network" [27] and the fusion layer by Feichtenhofer et al. [25]:

$$\hat{\mathbf{Z}}_V(i,j) = \mathbf{Z}_V(i,j) \cdot K + b. \tag{5}$$

The $1 \times 1$ convolution operation in Eq 5 uses a stride $s = 1$, convolution kernels $K$ of shape $1 \times 1 \times Dn_V \times D$ and bias terms $b \in \mathbb{R}^D$. The kernels contain trainable weights for computing weighted linear combinations of all $Dn_V$ input channels and are shared for all spatial positions $(i, j)$. The kernel weights are optimized by applying gradient descent for the objective of minimizing the classification loss, hence in the same way as any other trainable weights of the CNN. Early fusion (conv) preserves the correspondence between feature maps of the different views. Even more, the $1 \times 1$ convolution kernels will be trained for approximating the best weighted combination of feature maps across all views. However, this comes at cost of increasing model size, especially if the view fusion layer is inserted deep into the network. For example, with an input image shape of $224 \times 224 \times 3$ the feature maps returned by block 3 of the ResNet-50 have a dimensionality of $7 \times 7 \times 2,048$, i.e., $D = 2,048$ channels. Concatenation of feature maps from three views creates a tensor with 6,144 channels. Hence, the kernel size of the view fusion layer amounts to $1 \times 1 \times 6,144 \times 2,048$, i.e., 12,460,032 weights and 2,048 bias values. In contrast, performing fusion early in the network requires less trainable parameters, e.g., after block 0, the kernel size of the view fusion layer will be $1 \times 1 \times 768 \times 256$, hence 196,864 trainable parameters. However, early convolution features will only encode images' low-level concepts and likely result in limited classification accuracy.

We systematically vary the position of the view-fusion layer for the early fusion (max) and the early fusion (conv) variants. In detail, we sequentially incorporate the view-fusion layer at four different positions in between the main blocks of the ResNet architecture (cp. Fig 2c). The output $\hat{\mathbf{Z}}_V$ of the view fusion layer is then fed into $NN_2$, which contains all remaining layers of the split original CNN.

## Late fusion

In contrast to convolutional feature maps in early fusion, **late fusion** is performed using the feature vector

$$\mathbf{z}^{(v)} = NN_1(\mathbf{x}^{(v)}) \in \mathbb{R}^{1 \times D}, \tag{6}$$

of the network's penultimate layer as image representation $\mathbf{z}^{(v)}$ (cp. Fig 2b). $NN_2$ consists then merely of the classifier part of the original CNN. In case of the ResNet, the classifier part is composed of one one fully connected layer with softmax activation. We consider two late fusion approaches: (1) **late fusion (max)**: max-pooling of the stacked feature vectors across the $n_V$ views, and (2) **late fusion (fc)**: concatenation and fully connected fusion.

For **late fusion (max)** we stack the view's $n_V$ feature vectors, analogous to the multi-view CNN proposed by Su et al. [11], as

$$\boldsymbol{Z}_V = [\boldsymbol{z}^{(1)}; \ldots; \boldsymbol{z}^{(n_V)}] \in \mathbb{R}^{1\times D\times n_V} \tag{7}$$

and apply max-pooling across them as defined in Eq 4. Again, this operation sacrifices the correspondence between the different views.

For **late fusion (conv)**, we concatenate the feature vectors of the $n_V$ branches, analogous to [17, 21, 23, 24], as

$$\boldsymbol{Z}_V = [\boldsymbol{z}^{(1)}, \ldots, \boldsymbol{z}^{(n_V)}] \in \mathbb{R}^{1\times Dn_V}, \tag{8}$$

where $D$ is the length of each feature vector The resulting combined representation is then used as input for a fully connected layer of 1,024 neurons with ReLU activation and 50% dropout probability. This layer learns linear combinations of the features of all views in order to minimize classification loss. Finally, the fused representation $\hat{\boldsymbol{Z}}_V$ is used as input for $NN_2$ that merely consists of the last fully connected layer with softmax activation. Analogous to early fusion (conv), the late fusion (fc) strategy introduces trainable parameters in the view fusion layer. For the ResNet-50 architecture, the penultimate layer is a global average pooling layer that returns feature vectors having 2,048 dimensions. Concatenation of these feature vectors for, e.g., three views creates a vector of 6,144 dimensions, which is fully connected to the 1,024 neurons of the view fusion layer. Hence, the view fusion layer for the late fusion (fc) of three views contains 6,292,480 trainable parameters. In general, the view fusion layer for late fusion (fc) requires half the amount of trainable parameters compared to an early fusion (conv) after block 3.

## Score fusion

**Score fusion** is performed by element-wise aggregation of the softmax classification scores $y^{(v)}$ per image view $\boldsymbol{x}^{(v)} \in \boldsymbol{X}_V$ separately propagated through NN (cp. Fig 1b). We study the following aggregation functions [19, 22]:

- **sum-score fusion**: summation of scores across views

$$y_{V,\oplus} = \sum_v y^{(v)}; \tag{9}$$

- **product-score fusion**: multiplication of scores across views

$$y_{V,\otimes} = \prod_v y^{(v)}; and \tag{10}$$

- **max-score fusion**: maximum of scores across views

$$y_{V,\max} = \max_v y^{(v)}. \tag{11}$$

## Experimental procedure

We evaluate these fusion strategies and their respective aggregation and fusion operations in multi-view classification tasks of image collections. The entirety of all fusion strategies spawns a series of 14 experiments per dataset, i.e., 8× early fusion, 2× late fusion, 3× score fusion, and single-view classification as baseline. In order to systematically compare the performance of the different strategies we applied the following experimental procedure per dataset:

1. Training of a general-purpose single-view CNN on randomly shuffled batches of the entire training data. The single-view CNN acts as baseline for each experiment.
2. Initialization of the multi-view network by duplication of the $NN_1$ part per view and addition of the respective view fusion layer and remaining $NN_2$. The $NN_1$ and $NN_2$ parts are re-used from the single-view classifier.
3. Freezing of all $NN_1$ branches to ensure branches extract same features independent of the fusion strategy. This ensures that any effect on classification performance results solely from the fusion strategy.
4. Training of the multi-view networks on the multi-view image collections by optimizing the weights of the view fusion layer and $NN_2$.

Except for the size of the minibatches, we used the same hyper-parameters for all classifiers. Classification loss is computed as categorical cross-entropy loss and optimization is performed using Adam optimizer with an initial learning rate of 1e-4. Training was stopped once the validation loss stopped decreasing for ten epochs. For singe-view classification, we used a minibatch size of $bs_0 = 32$ and trained the CNN by randomly alternating between images of different views. Compared to corresponding single-view datasets, the size of each multi-view dataset is factorized by the number of views. That is, a multi-view classifier for two views processes twice as many training images per minibatch than a single-view classifier. Hence, for multi-view fusion, training was performed on minibatches with size $bs_0/n_V$ ensuring a constant number of images per batch. Each single-view CNN was fine-tuned after initialization with weights obtained from pre-training on the ILSVRC dataset [2]. To mitigate class-imbalance, the loss per sample was weighted with the inverse normalized class frequency of the corresponding class. All experiments were conducted using Keras v2.1.6 [28] with TensorFlow v1.8 backend [29].

## Datasets and evaluation

### Datasets

We evaluate the proposed view-fusion strategies on three benchmark datasets from different domains. These datasets represent fine-grained classification problems, i.e., they are characterized by large inter-class visual similarity and low intra-class similarity. An example collection of each dataset is shown in Fig 3. The **CompCars** dataset [30] contains web-nature and

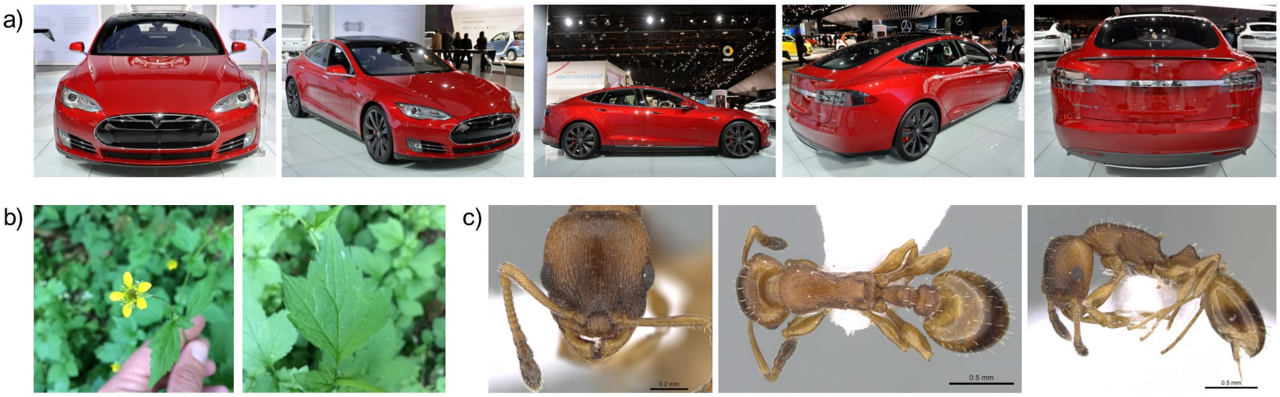

**Fig 3. Example collections of the three multi-view datasets: (a) CompCars, (b) PlantCLEF, and (c) AntWeb.** Photographs of the ant specimen CASENT0281563 by Estella Ortega retrieved from www.AntWeb.org [32].

https://doi.org/10.1371/journal.pone.0245230.g003

surveillance-nature images of cars. Web-nature images are annotated with car make, car model, year, and viewpoint. Each viewpoint is one of front, front-side, rear, rear-side, or side. Per car model and year, we sample one image per viewpoint for obtaining collections. We retain 601 car models represented by ≥10 collections irrespective of the model year resulting in 8,183 collections in total. Since we had to ignore the model year in favor of sufficient training data, car models may be tested using imagery of model years not part of the training data. The **PlantCLEF 2016** dataset [10] consists of observations of plant specimen and provides annotations in terms of organ, species, genus, and family. We select the subset of 1,839 collections that contain one image of the flower and one of the leaf. In order to have sufficient training data, i.e., ≥20 collections per class, we aggregate species observations and use the plant genus as class label. **AntWeb** [31] is an online database on ant biology providing structured image collections for ant species. Per specimen, we follow the procedure described by Marques et al. [5] and sample one image per dorsal, head, and profile view as one collection and retain 82 ant genera represented by ≥100 collections.

## Dataset demographics

Table 2 compares the three datasets in terms of descriptive statistics. In order to assess and compare the difficulty of the classification task associated with each dataset, we compute intra- and inter-class distances across each datasets' images. In detail, we compute the nearest neighbor Euclidean distance

$$d_i = \min_{j \neq i} \sqrt{(\boldsymbol{z}_i - \boldsymbol{z}_j)} \tag{12}$$

between the image representation $\boldsymbol{z}_i$ of the $i$-th sample and the representations $\boldsymbol{z}_j$ of all other samples $j$ belonging to the same class. The class-averaged mean across all samples per class is reported as intra-class distance in Table 2. Likewise, we compute the inter-class distance to all samples of any other class than the one of the $i$-th sample. Furthermore, we compute the silhouette score, an established metric comparing intra-class tightness to inter-class separation [33]. The silhouette of the $i$-th sample is defined as

$$s_i := \frac{b_i - a_i}{\max(a_i, b_i)}, \tag{13}$$

**Table 2. Dataset demographics.** Top-1 accuracy refers to the best reported result in previous single-view studies using comparable evaluation protocols.

| Dataset | PlantCLEF | CompCars | AntWeb |
|---|---|---|---|
| Number of images | 3,678 | 40,915 | 116,742 |
| Number of classes | 53 | 601 | 82 |
| Number of views | 2 | 5 | 3 |
| Number of collections | 1,839 | 8,183 | 38,914 |
| Intra-class distance | 23.24 | 19.11 | 21.99 |
| Inter-class distance | 24.54 | 21.26 | 23.02 |
| Distance ratio | 0.95 | 0.9 | 0.96 |
| Silhouette score | -0.003 | -0.014 | -0.013 |
| Previous top-1 accuracy | 85.9%[1] [3] | 76.7%[2] [30] | 83%[3] [5] |

[1] Evaluated across 516 plant genera (117,713 images) using Inception-ResNet-v2 network.

[2] Evaluated across 431 car models (30,955 images) using Overfeat network.

[3] Evaluated across 57 ant genera (128,832 images) using an ensemble of seven AlexNet networks.

https://doi.org/10.1371/journal.pone.0245230.t002

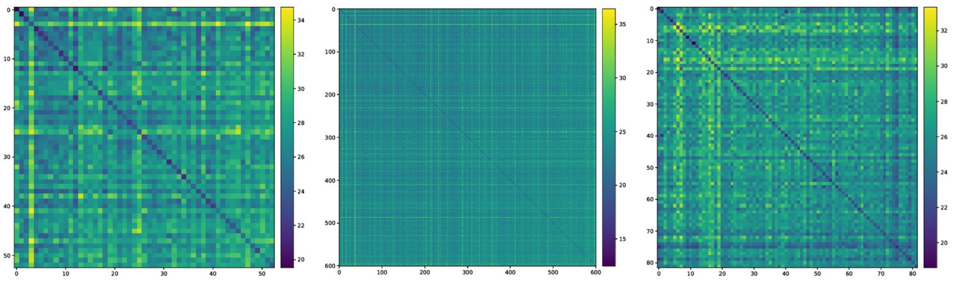

(a) PlantCLEF (b) CompCars (c) AntWeb

**Fig 4. Distance matrices for the three datasets.** Matrix diagonal elements refer to intra-class distance, off-diagonal elements to inter-class distances. Elements are sorted from well-separable classes to less-separable classes as computed from the class-wise silhouette scores.

https://doi.org/10.1371/journal.pone.0245230.g004

where $a_i$ is the average Euclidean distance between the representations $\boldsymbol{z}_i$ of the $i$-th sample and the representations $\boldsymbol{z}_j$ of all other images belonging to the same class as the $i$-th sample and $b_i$ is the average Euclidean distance between the $i$-th sample and all representations $\boldsymbol{z}_j$ of the closest different class. The silhouette score in Table 2 is then computed as class-averaged mean across all samples. Fig 4 shows distance matrices ordered by decreasing silhouette score per class providing a visual overview across the datasets.

Previous studies demonstrated that pre-trained CNNs are well-suited for extracting generic but discriminant image representations for downstream tasks and transfer-learning approaches [34]. Hence, we infer all representations $\boldsymbol{z}$ as output of the mean average pooling layer of a standard single-view ResNet-50 trained on the ImageNet ILSVRC dataset.

Apart from differing in the number of images, classes, and views, all datasets share high intra- to inter-class distance ratios, i.e., 0.95 for PlantCLEF, 0.9 for CompCars, and 0.96 for AntWeb. Such high ratios indicate a high inter-class resemblance accompanied by high intra-class visual variability. Also the low silhouette scores show that the attribution of images to their respective classes based on generic image representations is not distinct, and that samples of other classes are visually very close. We attribute the low silhouette scores to the different types of views these datasets contain. For example, different car models imaged from the side are visually closer to each other compared to images of the same car model imaged from the side and the front. Hence, low silhouette scores indicate that different views within a collection contribute complementary visual information making the three datasets perfectly suited for our study.

## Evaluation protocol and metrics

Per dataset, we use 80% of the collections per class for training the single-view and multi-view classifiers. The remaining 20% are used for testing and evaluation. We evaluate all experiments in terms of top-1 and top-5 accuracy, averaged across all collections of the respective test dataset. We compute top-$k$ accuracy as fraction of test collections where the ground-truth class label appears in the list of the first $k$ predicted class labels when predictions are sorted by decreasing classification score.

# Results and discussion

In this section, we compare the results of our multi-view classification strategies against single-view baseline experiments as well as against results of previous studies proposing multi-view

**Table 3. Multi-view classification results across the three datasets.**

| Method | Layer | top-1 [%] & $\delta_{BL}$ [%] | | | | | |
|---|---|---|---|---|---|---|---|
| | | PlantCLEF | | CompCars | | AntWeb | |
| Worst single view | | 65.80 | | 65.29 | | 79.34 | |
| Best single view | | 81.32 | | 82.33 | | 87.65 | |
| Avg. across single views | | 73.56 | | 76.61 | | 84.58 | |
| Early (max) | block 0 | 65.80 | -19.1 | 53.91 | -34.5 | 77.79 | -11.2 |
| | block 1 | 70.98 | -12.7 | 73.95 | -10.2 | 85.65 | -2.3 |
| | block 2 | 83.62 | 2.8 | 93.64 | 13.7 | 89.73 | 2.4 |
| | block 3 | 85.34 | 4.9 | 95.11 | 15.5 | 92.28 | 5.3 |
| Early (conv) | block 0 | 42.82 | -47.3 | 54.68 | -33.6 | 87.31 | -0.4 |
| | block 1 | 57.76 | -29.0 | 74.44 | -9.6 | 90.51 | 3.3 |
| | block 2 | 79.60 | -2.1 | 94.13 | 14.3 | 92.35 | 5.4 |
| | block 3 | 89.37 | 9.9 | **95.11** | **15.5** | **95.16** | **8.6** |
| Late (max) | fc | **90.23** | **11.0** | 92.82 | 12.7 | **93.93** | **7.2** |
| Late (fc) | fc | **94.25** | **15.9** | **96.72** | **17.5** | **94.54** | **7.9** |
| Score (⊗) | softmax | **89.66** | **10.3** | **95.74** | **16.3** | 91.43 | 4.3 |
| Score (⊕) | softmax | 86.78 | 6.7 | 94.69 | 15.0 | 89.70 | 2.3 |
| Score (max) | softmax | 85.34 | 4.9 | 92.88 | 12.8 | 89.48 | 2.1 |

Best methods are highlighted in 1st—green, 2nd—light green, 3rd—gray green font color. Red values indicate results worse compared to baseline results. Single view accuracy results refer to the worst performing single view, the best performing single view, as well as to the average across all available views. $\delta_{BL}$ is calculated as relative difference to the best single view result.

https://doi.org/10.1371/journal.pone.0245230.t003

classification. Our results across all datasets for all single view and view-fusion experiments are summarized in Table 3. The distribution of class-averaged classification accuracy for the different experiments is visualized in Fig 5.

Table 2 shows previous results across the three utilized datasets allowing for a comparison with our results. In contrast to previous results we yield a lower accuracy on the PlantCLEF dataset, which we attribute to the small number of training images (2,982 for 53 classes) in our experiments as we had to restrict data to images in collections, while previous results were based on the 32× bigger entire training set at genus level [3]. On the CompCars dataset, our single view predictions yield the same top-1 accuracy as reported before by Yang et al. [30]. Please note that we classify among 601 car models rather than the reduced 431 models [30]. On the AntWeb dataset, our single view predictions slightly outperform the accuracy reported by Marques et al. [5]. They used an ensemble of seven AlexNet models for classifying 57 ant genera [5]. In our experiments, classification was performed across 82 ant genera. Overall, we find average accuracy of the single view predictions to be plausible and comparable to previously reported results demonstrating the validity of our study.

Accuracy yielded by the different fusion strategies differs substantially in contrast to the best single-view baseline. In detail, we observe that the accuracy of **early fusion** depends on dataset size, especially if fusion is performed by 1 × 1 convolutions (cp. Fig 2b). If fusion is performed early in the network, i.e., after block 0 and block 1, we found accuracy to be reduced for all datasets in comparison to single view classification. However, the relative distances to the best single view decreases with increasing dataset size. Early fusion of block 3 features by 1 × 1 convolution achieved the highest average classification accuracy on the AntWeb dataset. However, the number of misclassified classes is larger compared to late fusion (cp. with Fig 5). The late fusion strategies generally allow for higher classification

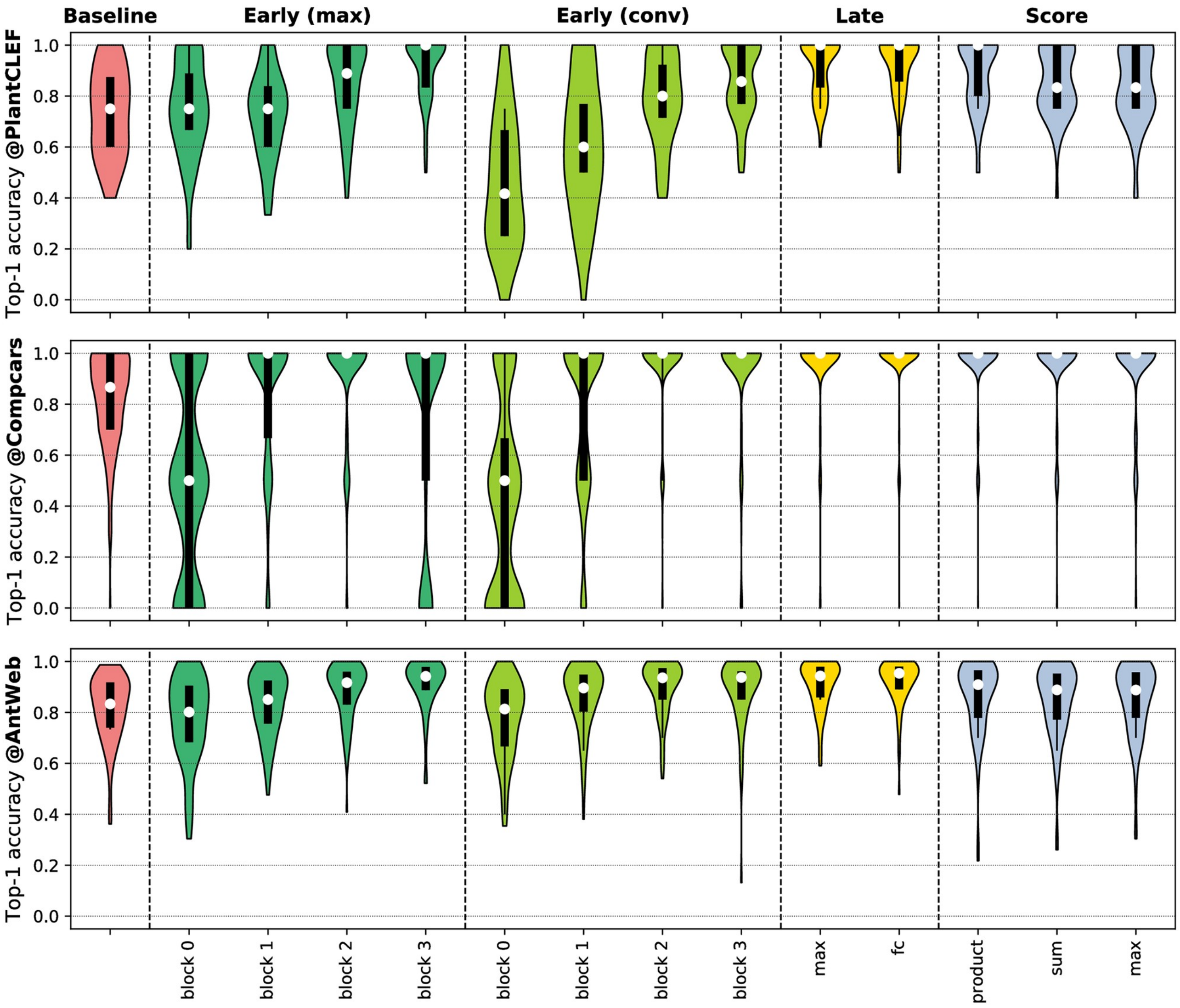


**Fig 5. Distribution of class-averaged top-1 classification accuracy for the single-view baseline and the multi-view classification strategies.** White dots indicate median accuracy whereas black bars display interquartile ranges. Thin black lines indicate lower and upper adjacent values at 1.5× the interquartile range.

https://doi.org/10.1371/journal.pone.0245230.g005

accuracy compared to single view experiments. Product-score fusion also allows for notable improvements (10.3% on average) compared to the best single view predictions. Do et al. also found product-score fusion to yield the highest classification accuracy in score fusion experiments [19]. Comparing the late fusion strategies with each other, we conclude that the element-wise max-operation on the image representations tend to confuse the classifier once the number of views increases. In detail, on the CompCars dataset with five views per collection, classification accuracy of the late fusion (max) strategy reduces by -3.9% compared to the accuracy achieved by late fusion (fc). Overall, the improvement in average classification accuracy by the late fusion (fc) strategy was either the largest or among the largest. The distribution of average classification accuracy per class in Fig 5 shows, that the median of the accuracy was the largest for late fusion (fc) and that the number of outlier classes was smaller compared to other strategies.

**Table 4. Top-5 accuracy for single-view and multi-view classifications.**

| Method | top-5 [%] | | |
|---|---|---|---|
| | PlantCLEF | CompCars | AntWeb |
| Avg. across single views | 93.39 | 91.56 | 97.31 |
| Best single view | 96.26 | 94.48 | 98.31 |
| Late (fc) | 99.71 | 99.02 | 99.29 |

https://doi.org/10.1371/journal.pone.0245230.t004

Table 4 shows top-5 classification accuracy for single- and multi-view classifications fused by late (fc) fusion. Across datasets, for more than 99% of the test collections, the correct class was within the top five predicted classes when using multi-view classification.

Considering dataset characteristics, the CompCars dataset has the smallest silhouette score (cp. Table 2), indicating large visual variations across different views. Likewise, the relative improvement compared to the best single view predictions is the highest (17.5%) for the CompCars dataset. Some collections of the CompCars dataset were misclassified irrespective of the classification and fusion strategy (cp. with outliers in Fig 5). We attribute this to model years that were only part of the test set. The relative improvement was the smallest (8.6%) on the AntWeb dataset, which is characterized by the largest intra- vs. inter-class distance ratio among the three datasets.

## Application scenario: Plant species identification

The results in Section *Results and Discussion* show that the late fusion (fc) strategy displays the best compromise in classification accuracy. As this strategy is entirely independent from the underlying network architecture, it can easily be used for extending other network architectures for computing fused image representations (cp. Sec. *Fusion Strategies for Multi-view Image Classification*). In this section, we aim to demonstrate the applicability and accuracy gain that this multi-view classification strategy provides when applied to an existing and fully trained single-view CNN. We use an existing classification model trained for the Flora Incognita project [35]. The single view classifier uses a NASNet-A architecture [36] trained with 1.3 million images of wild-flowering plants growing in Germany. The data was collected from web resources as well as through a citizen-science initiative using the Flora Capture [37] and Flora Incognita smartphone applications [35]. These apps prescribe a collection-based sampling strategy, i.e., every plant specimen is captured by multiple images depicting distinct plant organs or perspectives. In total, 8,557 test image collections confirmed by human expert were available at the time of this study. These collections represent 775 distinct plant species by side-view images of their flowers and top-view images of their leaves (cp. Table 5). The intra- vs. inter-class distance ratio of this dataset increased to 1.02 compared to the other datasets, indicating very close visual resemblance of different plant species and a fine-grained classification problem.

Following the procedure described in Section *Fusion Strategies for Multi-view Image Classification*, we used the trained single-view NASNet-A and extracted image representations from the global average pooling layer prior to the fully connected classification layer. For each image, a center crop retaining 50% of an image's area was resized to 299 × 299 px and forwarded through the network. Per image, only one representation was computed from the center crop. Next, we constructed view-fusion networks consisting of a view fusion layer of the respective strategy and a classification layer. We evaluate both late fusion strategies, i.e., by max-pooling across views as well as concatenation and dense connection. In addition, we evaluate score fusion defined in Eqs 9–11 using the softmax class scores obtained by the single-

**Table 5. Dataset demographics for the Flora Incognita dataset.**

| Dataset | Flora Incognita |
|---|---|
| Number of images | 17,114 |
| Number of classes | 775 |
| Number of views | 2 |
| Number of collections | 8,557 |
| Intra-class distance | 25.26 |
| Inter-class distance | 24.70 |
| Distance ratio | 1.02 |
| Silhouette score | -0.014 |

https://doi.org/10.1371/journal.pone.0245230.t005

**Table 6. Multi-view classification results for the Flora Incognita dataset.**

| Method | top-1 [%] | $\delta_{BL}$ [%] |
|---|---|---|
| Worst single view | 86.03 | |
| Best single view | 89.74 | - |
| Avg. across single views | 87.87 | |
| Late (max) | 94.10 | 4.9 |
| Late (fc) | 96.07 | 7.1 |
| Score (⊗) | 94.54 | 5.3 |
| Score (⊕) | 93.67 | 4.4 |
| Score (max) | 93.01 | 3.6 |

https://doi.org/10.1371/journal.pone.0245230.t006

view baseline model. The results of the single-view baseline and the multi-view networks are summarized in Table 6 in terms of top-1 accuracy and relative improvement against the best single-view accuracy. Single-view accuracy amounts to 89.74% for the best view. Applying the late fusion (fc) strategy, classification accuracy increased to 96.07%.

## Conclusion

We report a systematic evaluation of fusion strategies for multi-view image classification using convolutional neural networks. Our results on three datasets from different domains show that classification accuracy increases if fusion of latent representations is performed late in the network. At cost of increased model size, trainable view fusion was generally found more accurate compared to fusion by max-pooling of latent representations or score fusion using different arithmetics. Among trainable view fusion strategies, late fusion by feature vector concatenation in combination with one fully connected layer yields the largest increase in classification accuracy and requires merely half the parameters compared to deep feature map fusion by $1 \times 1$ convolution. Furthermore, we demonstrate applicability and accuracy gain of late view fusion by successfully transforming an already trained single-view NASNet-A model into a multi-view classifier thereby gaining a substantial accuracy improvement.

## Author Contributions

**Conceptualization:** Marco Seeland.

**Funding acquisition:** Patrick Mäder.

**Investigation:** Marco Seeland.

**Methodology:** Marco Seeland.

**Project administration:** Patrick Mäder.

**Resources:** Patrick Mäder.

**Software:** Marco Seeland.

**Visualization:** Marco Seeland.

**Writing – original draft:** Marco Seeland.

**Writing – review & editing:** Marco Seeland, Patrick Mäder.

## References


1. LeCun Y, Bengio Y, Hinton G. Deep Learning. Nature. 2015; 521(7553):436–444. https://doi.org/10.1038/nature14539 PMID: 26017442
2. Russakovsky O, Deng J, Su H, Krause J, Satheesh S, Ma S, et al. ImageNet Large Scale Visual Recognition Challenge. International Journal of Computer Vision. 2015; 115(3):211–252. https://doi.org/10.1007/s11263-015-0816-y
3. Seeland M, Rzanny M, Boho D, Wäldchen J, Mäder P. Image-based classification of plant genus and family for trained and untrained plant species. BMC Bioinformatics. 2019; 20(1):4. https://doi.org/10.1186/s12859-018-2474-x PMID: 30606100
4. Wäldchen J, Rzanny M, Seeland M, Mäder P. Automated plant species identification—Trends and future directions. PLOS Computational Biology. 2018; 14(4):1–19. https://doi.org/10.1371/journal.pcbi.1005993 PMID: 29621236
5. Marques ACR, Raimundo MM, Cavalheiro EMB, Salles LFP, Lyra C, Von Zuben FJ. Ant genera identification using an ensemble of convolutional neural networks. PLOS ONE. 2018; 13(1):1–13. https://doi.org/10.1371/journal.pone.0192011 PMID: 29385214
6. Połap D. Analysis of Skin Marks Through the Use of Intelligent Things. IEEE Access. 2019; 7:149355–149363. https://doi.org/10.1109/ACCESS.2019.2947354
7. Li Y, Yang M, Zhang ZM. A Survey of Multi-View Representation Learning. IEEE Transactions on Knowledge and Data Engineering. 2018; p. 1–1. https://doi.org/10.1109/TKDE.2018.2872063
8. Juočas L, Raudonis V, Maskeliūnas R, Damaševičius R, Woźniak M. Multi-focusing algorithm for microscopy imagery in assembly line using low-cost camera. The International Journal of Advanced Manufacturing Technology. 2019; 102(9-12):3217–3227. https://doi.org/10.1007/s00170-019-03407-9
9. Li S, Song W, Qin H, Hao A. Deep variance network: An iterative, improved CNN framework for unbalanced training datasets. Pattern Recognition. 2018; 81:294—308. https://doi.org/10.1016/j.patcog.2018.03.035
10. Goëau H, Bonnet P, Joly A. Plant Identification in an Open-world (LifeCLEF 2016). In: CLEF 2016—Conference and Labs of the Evaluation forum. Évora, Portugal; 2016. p. 428–439. Available from: https://hal.archives-ouvertes.fr/hal-01373780.
11. Su H, Maji S, Kalogerakis E, Learned-Miller E. Multi-view Convolutional Neural Networks for 3D Shape Recognition. In: 2015 IEEE International Conference on Computer Vision (ICCV). vol. 00; 2015. p. 945–953. Available from: doi.ieeecomputersociety.org/10.1109/ICCV.2015.114.
12. Hofmann M, Seeland M, Mäder P. Efficiently Annotating Object Images with Absolute Size Information Using Mobile Devices. International Journal of Computer Vision. 2018;. https://doi.org/10.1007/s11263-018-1093-3
13. Połap D. Human-machine interaction in intelligent technologies using the augmented reality. Inf Technol Control. 2018; 47:691–703.
14. Žemgulys J, Raudonis V, Maskeliūnas R, Damaševičius R. Recognition of basketball referee signals from real-time videos. Journal of Ambient Intelligence and Humanized Computing. 2020; 11(3):979–991. https://doi.org/10.1007/s12652-019-01209-1
15. Muzammil SR, Maqsood S, Haider S, Damaševičius R. CSID: A Novel Multimodal Image Fusion Algorithm for Enhanced Clinical Diagnosis. Diagnostics (Basel, Switzerland). 2020; 10(11). https://doi.org/10.3390/diagnostics10110904 PMID: 33167376
16. Feng Y, Zhang Z, Zhao X, Ji R, Gao Y. GVCNN: Group-View Convolutional Neural Networks for 3D Shape Recognition. In: The IEEE Conference on Computer Vision and Pattern Recognition (CVPR); 2018.

17. Lin C, Kumar A. Contactless and partial 3D fingerprint recognition using multi-view deep representation. Pattern Recognition. 2018; 83:314—327. https://doi.org/10.1016/j.patcog.2018.05.004

18. Wang A, Cai J, Lu J, Cham TJ. MMSS: Multi-Modal Sharable and Specific Feature Learning for RGB-D Object Recognition. In: The IEEE International Conference on Computer Vision (ICCV); 2015.

19. Do T, Nguyen H, Nguyen T, Vu H, Tran T, Le T. Plant identification using score-based fusion of multi-organ images. In: 2017 9th International Conference on Knowledge and Systems Engineering (KSE); 2017. p. 191–196.

20. Lee SH, Chan CS, Remagnino P. Multi-Organ Plant Classification Based on Convolutional and Recurrent Neural Networks. IEEE Transactions on Image Processing. 2018; 27(9):4287–4301. https://doi.org/10.1109/TIP.2018.2836321 PMID: 29870348

21. Setio AAA, Ciompi F, Litjens G, Gerke P, Jacobs C, van Riel SJ, et al. Pulmonary Nodule Detection in CT Images: False Positive Reduction Using Multi-View Convolutional Networks. IEEE Transactions on Medical Imaging. 2016; 35(5):1160–1169. https://doi.org/10.1109/TMI.2016.2536809 PMID: 26955024

22. Dolata P, Mrzygłód M, Reiner J. Double-stream Convolutional Neural Networks for Machine Vision Inspection of Natural Products. Applied Artificial Intelligence. 2017; 31(7-8):643–659. https://doi.org/10.1080/08839514.2018.1428491

23. Barbosa A, Marinho T, Martin N, Hovakimyan N. Multi-Stream CNN for Spatial Resource Allocation: A Crop Management Application. In: Proceedings of the IEEE/CVF Conference on Computer Vision and Pattern Recognition (CVPR) Workshops; 2020.

24. Geras KJ, Wolfson S, Shen Y, Wu N, Kim SG, Kim E, et al. High-Resolution Breast Cancer Screening with Multi-View Deep Convolutional Neural Networks; 2017.

25. Feichtenhofer C, Pinz A, Zisserman A. Convolutional Two-Stream Network Fusion for Video Action Recognition. In: 2016 IEEE Conference on Computer Vision and Pattern Recognition (CVPR); 2016. p. 1933–1941.

26. He K, Zhang X, Ren S, Sun J. Deep Residual Learning for Image Recognition. In: 2016 IEEE Conference on Computer Vision and Pattern Recognition (CVPR); 2016. p. 770–778.

27. Lin M, Chen Q, Yan S. Network In Network. CoRR. 2013;abs/1312.4400.

28. Chollet F, et al. Keras; 2015. https://github.com/fchollet/keras.

29. Abadi M, Barham P, Chen J, Chen Z, Davis A, Dean J, et al. TensorFlow: A System for Large-Scale Machine Learning. In: 12th USENIX Symposium on Operating Systems Design and Implementation (OSDI 16). Savannah, GA: USENIX Association; 2016. p. 265–283.

30. Yang L, Luo P, Loy CC, Tang X. A large-scale car dataset for fine-grained categorization and verification. In: 2015 IEEE Conference on Computer Vision and Pattern Recognition (CVPR); 2015. p. 3973–3981.

31. California Academy of Sciences. Antweb; 2019.

32. Ortega E. CASENT0281563 from https://www.antweb.org; 2020.

33. Rousseeuw PJ. Silhouettes: A graphical aid to the interpretation and validation of cluster analysis. Journal of Computational and Applied Mathematics. 1987; 20:53—65. https://doi.org/10.1016/0377-0427(87)90125-7

34. Sharif Razavian A, Azizpour H, Sullivan J, Carlsson S. CNN Features Off-the-Shelf: An Astounding Baseline for Recognition. In: The IEEE Conference on Computer Vision and Pattern Recognition (CVPR) Workshops; 2014.

35. Flora Incognita. The Flora Incognita Research Project; 2019.

36. Zoph B, Vasudevan V, Shlens J, Le QV. Learning Transferable Architectures for Scalable Image Recognition. CoRR. 2017;abs/1707.07012.

37. Boho D, Rzanny M, Wäldchen J, Nitsche F, Deggelmann A, Wittich HC, et al. Flora Capture: a citizen science application for collecting structured plant observations. BMC Bioinformatics. 2020; 21(576):4. https://doi.org/10.1186/s12859-020-03920-9 PMID: 33317442
